# Patient preference for intraoperative opioid use and early recovery after noncardiac surgery: protocol for a randomised factorial design trial of opioid-free *versus* opioid-based anaesthesia (the PERFECT trial)

**DOI:** 10.1016/j.bjao.2025.100420

**Published:** 2025-06-18

**Authors:** Yann Gricourt, Nancy M. Boulos, Amelie Delaporte, Brenton Alexander, Stephane Besada, Ryan Bakhit, Aline Toukhtarian, Ido Neuman, Daniel Pearce, Meziar M. Nourian, Arthur Chebishian, Amy Zhou, Janice Boktor, Dylan Mayanja, Tristan Grogan, David Boldt, Maxime Cannesson, Patrice Forget, Alexandre Joosten

**Affiliations:** 1 Department of Anesthesiology and Perioperative Medicine, David Geffen School of Medicine, University of California Los Angeles, CA, USA; 2 Department of Anesthesiology and Perioperative Medicine, Nimes University Hospital, Nimes, France; 3 IMAGINE UR UM 103, Montpellier University, Anesthesia Critical Care, Emergency and Pain Medicine Division, Nîmes University Hospital, Nîmes, France; 4 Department of Anesthesiology, UC San Diego School of Medicine, La Jolla, CA, USA; 5 Department of Anesthesiology, University of California Los Angeles, CA, USA; 6 Georgetown University School of Medicine, Washington, DC, USA; 7 Charles R. Drew University of Medicine and Science, Los Angeles, CA, USA; 8 Aberdeen Centre for Arthritis and Musculoskeletal Health (Epidemiology Group), Institute of Applied Health Sciences, School of Medicine, Medical Sciences and Nutrition, Aberdeen, UK; 9 Anaesthesia Department, NHS Grampian, Aberdeen, UK; 10 Pain and Opioids After Surgery (PANDOS) European Society of Anaesthesiology and Intensive Care (ID ESAIC_RG_PAND) Research Group, Brussels, Belgium

**Keywords:** minimally invasive surgical procedures, opioid-free anaesthesia, patient-centred care, quality of recovery, shared decision making

## Abstract

**Introduction:**

Although opioids are commonly used to relieve pain associated with surgery, they are not consequence free. Moreover, the USA and many western countries are currently experiencing a significant health crisis because of opioid addiction and its related overdose potential. There have been no studies that have evaluated patient preference regarding opioid use and its potential impact on the quality of recovery. The aim of this study is to compare the effect of patient preference on intraoperative opioid use on early postoperative quality of recovery after moderate risk laparoscopic/robotic abdominal surgery.

**Methods:**

This trial is an interventional, pragmatic, partially randomised factorial trial. Adults (*N*=240) scheduled for moderate-risk abdominal surgery under laparoscopic/robotic assistance (colorectal, urologic, and gynaecologic) will be allocated into four groups, according to their preference (choice of opioid-free *vs* opioid-based anaesthesia *vs* no choice and, if no choice, then the patient is randomised to opioid-based *vs* opioid-free anaesthesia). Anaesthesia providers and patients who choose their anaesthesia type will be unblinded of the allocation group. The primary endpoint will be the Quality of Recovery-15 score at postoperative day 1. Secondary endpoints will include patient satisfaction, postoperative nausea and vomiting, intraoperative bradycardia, postoperative opioid consumption, postoperative hypoxemia, and health-related quality of life using the EuroQoL 5-Dimension 5-Level (EQ-5D-5L).

**Conclusions:**

This trial will provide evidence on whether patient preference on intraoperative opioid use can improve patient quality of recovery after moderate-risk abdominal surgery.

**Clinical trial registration:**

NCT06855641.

**Protocol version number and date:**

2.0, 24 February 2025.

Involving patients in shared decision-making is widely recognised as a cornerstone of ideal patient care. In the context of the US opioid crisis, informing patients about their intraoperative opioid exposure (e.g. fentanyl, sufentanil, hydromorphone) is more relevant than ever. New anaesthesia techniques, including opioid-sparing or opioid-free anaesthesia (OFA), have emerged as alternatives to traditional opioid-based anaesthesia (OBA) to enhance patient outcomes and postoperative morbidity.

Given the fact that the total volume of minimally invasive surgeries has grown significantly in recent years^1^ and is now considered to be a gold standard approach in many cases, allowing patients to be informed of and participate in the selection of specific anaesthesia components could enhance patient autonomy and empowerment. This is particularly true as opioids are no longer a mandatory intraoperative component, and their use carries various risks. Opioids are associated with several side-effects, including nausea, vomiting, sedation, ileus, respiratory depression, hyperalgesia, addiction, and potential misuse.^2^ In response, current national and Enhanced Recovery After Surgery (ERAS) guidelines advocate for opioid-sparing approaches across various surgical models. Within this context, OFA has been proposed as an intraoperative opioid alternative, using techniques such as regional anaesthesia and non-opioid i.v. medications.^3^

OFA offers several potential advantages, including a reduction in postoperative nausea and vomiting (PONV)^4^^,^^5^ and improvements in postoperative recovery.^6^ However, in routine clinical practice, patients rarely express preferences regarding anaesthesia strategies, such as OFA or OBA, with the final decision typically made by clinicians. A recent US survey revealed that 43% of patients preferred a patient-led decision-making role, 28% preferred shared decision-making with their clinical team, and only 29% preferred a completely physician-led approach.^7^ Empowering patients and shared decision-making may impact resource allocation, care delivery and, ultimately, patient engagement in ERAS pathways. In the field of OFA research, recent studies have focused on early postoperative quality of recovery (QoR).^6 8^ However, no studies have previously evaluated the factors influencing a patient's choice between OFA and OBA, nor the impact of these choices on early QoR. Moreover, data on the potential impact of OFA on QoR in minimally invasive surgeries are lacking.

The aim of this pragmatic study is to compare the effect of patient preference regarding the anaesthesia strategy of OFA *vs* OBA after noncardiac surgery on early postoperative quality of recovery (QoR-15 score). We hypothesise patients who choose their anaesthesia strategy will have higher QoR-15 scores compared with patients whose anaesthesia type is determined randomly.

## Methods

### Study design and participants

#### Trial design

The PERFECT trial is an interventional prospective, pragmatic, controlled, partially randomised, blinded trial of superiority, conducted at two tertiary academic centres (Ronald Reagan and Santa Monica UCLA Medical Center) in Los Angeles, USA. Patient enrolment plan will take place between March 2025 and March 2026. This protocol was developed following the SPIRIT guidelines^9^ to ensure comprehensive reporting of interventional trial protocols (Supplementary File S1).

#### Participant selection and consent

According to inclusion criteria, the PERFECT trial investigators will identify eligible participants the day before surgery: participants will receive a phone call providing standardised information about protocol, OFA, and OBA. Written consent will be obtained on the day of surgery, before anaesthesia. Original protocols submitted to the institutional review board (IRB) and patient consent are given in Supplementary Files S2 and S3.

Inclusion criteria:

• Age >18 yr
• Undergoing elective intermediate risk surgery under general anaesthesia (robotic or laparoscopic-assisted urological, gynaecological or abdominal surgery), according to the 2022 European Society of Cardiology guidelines^10^
• American Society Anesthesiologists physical status classification system grades of 1 to 4
• English speaking
• Informed consent signed

Main exclusion criteria:

• Diagnosis of chronic pain
• Preoperative prescribed opioids
• Pregnancy or lactation
• History of mental disorders
• Contraindications to study drug (lidocaine, magnesium, dexmedetomidine, ketamine)
• Patient is participating in another interventional trial
• Patient is under judicial protection or is an adult under guardianship

## Allocation and blinding

### Factorial design details

**Fig 1:**
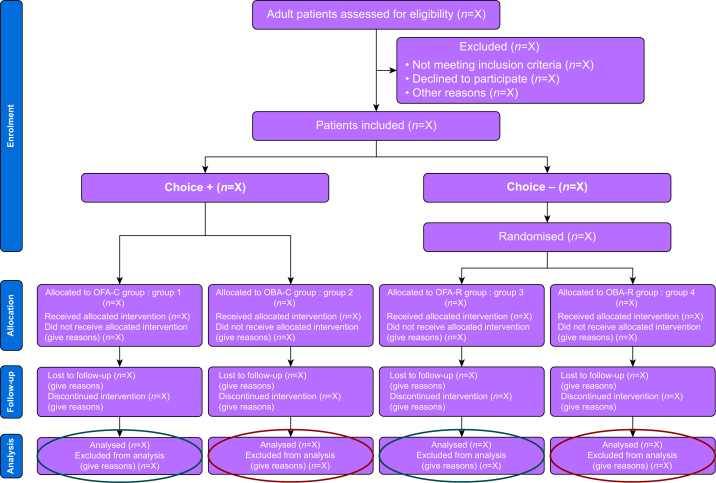
Study flow chart of the study design. C, choice; OBA, opioid-based anaesthesia; OFA, opioid-free anaesthesia; R, randomisation.

The study will include two key factors: patient choice (patient-led decision or a shared decision with anaesthesia team) and anaesthetic strategy type (OFA *vs* OBA) to explore their impacts on outcomes and potential interactions. Participants who decline to choose after receiving full information will be randomised to either OFA or OBA, whereas those who actively choose it will not be randomised. Consequently, the study follows a partially randomised factorial design. This design results in four study groups, as summarised in Figure 1.

In case of patients’ preference trial, randomised patient preference trials^11^ is a reliable alternative for randomised controlled trials and allows faster inclusion of a more representative population improving external validity without compromising internal validity.

### Patient distribution

• For the randomised groups 3 and 4 (OFA-R and OBA-R), a 1:1 allocation ratio will be used with a permuted block design (fixed block size of six). Each block will contain three patients assigned to OFA and three to OBA in a random order. A researcher not involved in patient care will generate the randomisation list using R V4.1.0 (‘blockrand’ package). The list will be stored in a secure file and provided to study coordinators in sequential order to assign each patient upon enrolment.
• For the choice groups 1 and 2, participants will not be randomised. Based on patient preferences, achieving an exact 1:1 ratio is unlikely. To address this, a capped enrolment strategy will be used. This approach ensures that enrolment into each choice group is monitored, and recruitment will stop for a group once its target size has been reached. This method minimises imbalance while respecting patient autonomy in selecting their preferred anaesthesia strategy.

### Partially blind status

The anaesthesia team for all groups performing the general anaesthesia and participants from groups 1 and 2 (OFA-C and OBA-C) will be unblinded on the allocation group whereas participants from groups 3 and 4 (OFA-R and OBA-R), surgeons, postoperative care providers, evaluators, and statisticians will all be blinded. To maintain the integrity of blinding, separate personnel will handle intraoperative (unblinded) and postoperative (blinded) care and evaluation. Additionally, postoperative recovery protocols will be standardised across all groups to minimise potential bias.

## Study interventions

The schedule of patient enrolment, study interventions, and outcome assessment are in accordance with the SPIRIT statement (Table 1).

**Table 1:**
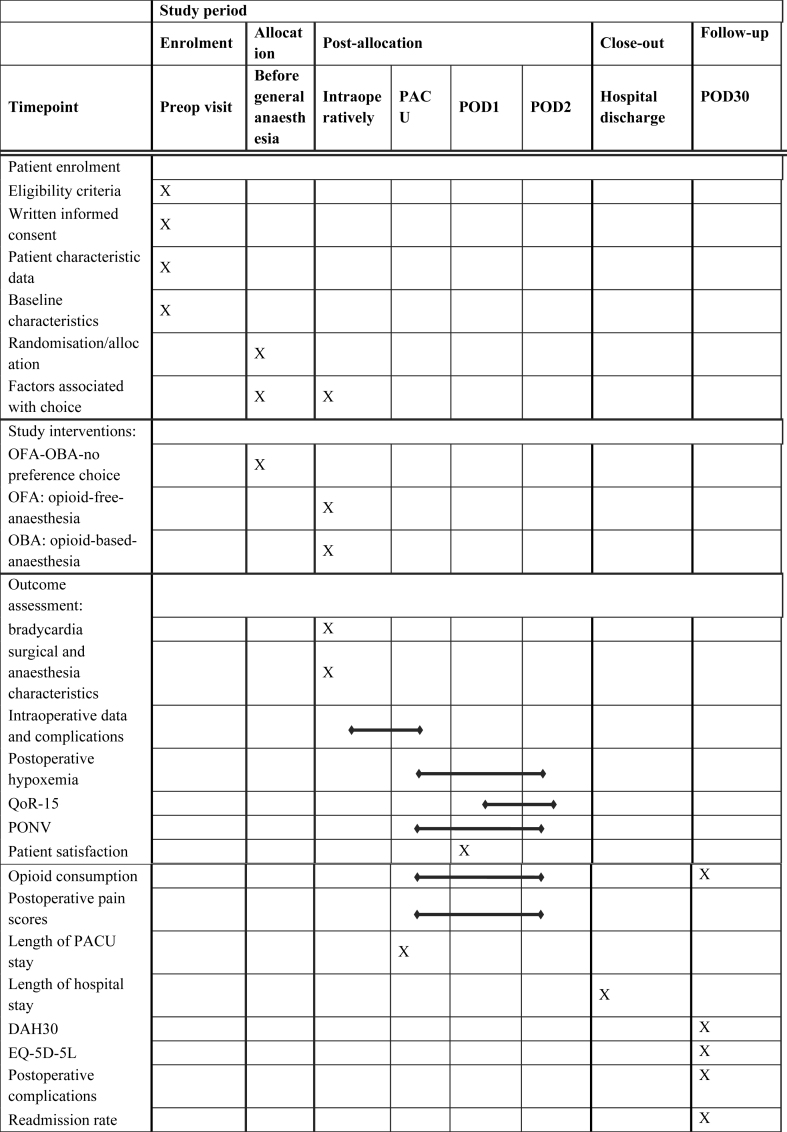
Schedule of enrolment, study interventions, and outcome assessments. According to SPIRIT statement of defining standard protocol items for clinical trials. DAH30, days at home 30; EQ-5D-5L, EuroQol 5 Dimension, five-level version with visual analogue scale; PACU, postoperative care unit; POD, postoperative day; PONV, postoperative nausea and vomiting; QoR-15, quality of recovery 15.

**Fig 2:**
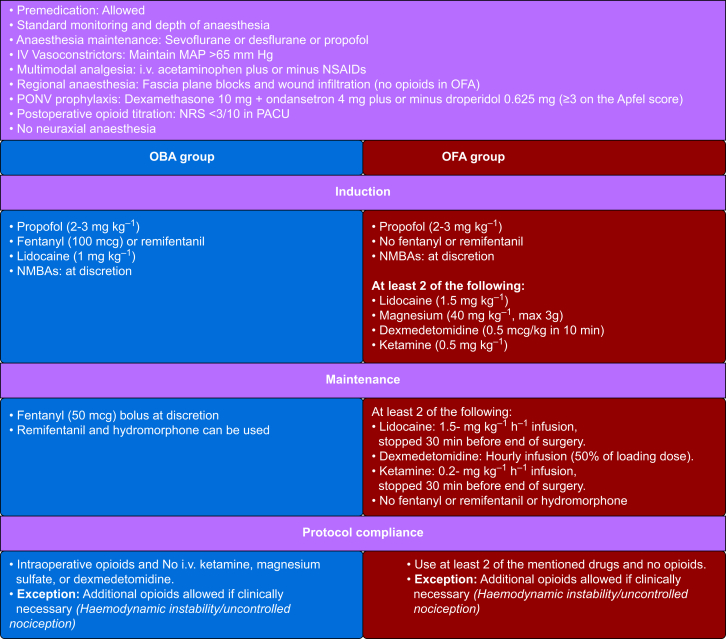
Study interventions—anaesthesia protocol. NMBAs, neuromuscular blocking agents; NRS, numeric rating scale; OBA, opioid-based anaesthesia; OFA, opioid-free anaesthesia; PONV, postoperative nausea and vomiting.

The day before surgery, participants will receive a phone call from PERFECT investigators to explain the study and provide standardised information about the benefits and drawbacks of OBA and OFA, and their options (choosing or randomisation). This information will follow a specific patient decision aid developed by the study team and patient partners. On the day of surgery, in the preoperative area, participants will decide whether to join the study and, if so, whether to potentially choose their anaesthesia strategy. Family members may assist in the decision if requested. Participants will also give feedback on the anaesthesia choice process. The study interventions are presented in Figure 2.

Compliance will be assessed from participants’ electronic medical records (EMR) and considered met regardless of specific doses or early infusion discontinuation. In case of haemodynamic instability or uncontrolled intraoperative nociception, anaesthesia providers will be allowed to administer i.v. opioids if deemed clinically necessary and relevant, regardless of the allocated group.

## Outcomes

All outcomes are summarised in Table 1. The total participation duration will be 30 days.

The primary endpoint will be the comparison of early postoperative QoR on postoperative day 1 (POD1) using the validated QoR-15 score (as a whole, and each item separately)^12^ between patients who choose *vs* do not choose their anaesthesia strategies.

The QoR-15 questionnaire will be filled by participants in the hospital or completed by the investigator via phone call if the patient has already been discharged. This score consists of 15 questions covering five key dimensions of postoperative recovery: physical comfort, emotional state, psychological support, physical independence, and pain management.

Secondary outcomes will include early postoperative QoR on POD1 depending on OFA or OBA, QoR on POD2, PONV incidence, anaesthesia satisfaction with Bauer questionnaire,^13^ postoperative opioid consumption, intraoperative bradycardia and postoperative hypoxaemia incidence, and health quality of life on POD30 with EQ-5D-5L score.^14^

Exploratory outcomes will include intraoperative measures and anaesthesia protocol adverse effects, time to awakening, length of PACU and hospital stays, postoperative pain intensity at rest, incidence of any postoperative surgical complication (Clavien–Dindo classification), and hospital readmission rate (%) both on POD30. Additionally, DAH30^15^ will be assessed as the number of days the patient is alive and at home within 30 days of the index surgery. It will be 0 if the patient dies within this period, regardless of time spent at home.

## Safety concerns

Adverse events will be recorded from anaesthesia induction to POD30 and included in the exploratory outcomes. In detail, participants will be continually monitored and under clinical supervision from anaesthesia induction to PACU discharge, and anaesthesia providers will be encouraged to report any adverse events related to the protocol. Management of it will follow current local practices.

Adverse events will be collected from EMR or during patient follow-up phone calls and reported to the PERFECT investigators. Upon hospital discharge, patients will receive contact information to report any potential adverse events. Any serious and unexpected adverse events associated with the use of the study drugs will be reported to the IRB and the Food and Drug Administration, without delay, by the primary investigators. Because of the bicentric design, intermediate risk participants, and selected minimal invasive surgeries, no data and safety monitoring board will be involved. However, an independent safety monitor from the same institution, not involved in the PERFECT trial, will be designated to monitor patient safety. UCLA will provide necessary medical treatment if any participant is injured as a result of being involved in the study.

## Data collection and monitoring

Data will be collected into a case report form by investigators blinded to the allocation group, using UCLA EMR or participant phone call.

All data will be securely stored on a password-protected server hosted at UCLA, in compliance with institutional data security policies and regulatory requirements. Access to the data will be restricted to authorised study personnel only. Each participant will be assigned a unique study identification number to ensure confidentiality and anonymity.

All participant collected data are summarised in Table 1. Patient characteristic data, medical history, specific factors associated with the anaesthesia choice (history of opioid use or addiction, smoking status, substance or drug abuse, history of chronic pain, prior PONV, previous abdominal surgeries, and type of scheduled surgery), and anaesthesia choice process data will be initially recorded. Comorbidities include cardiovascular, respiratory, neurological, kidney and liver dysfunction or cancer. Surgical and anaesthesia characteristics, intraoperative data, and all complications are linked to exploratory outcomes. Data of the study, including consent forms, will be kept for 15 yr after the study completion in a dedicated secure server.

## Sample size calculation

Based on a previous and similar study^8^ with a comparable anaesthesia protocol and inclusion criteria, the mean QoR-15 score at 24 h was 114.9 (15.2) in the OFA group *vs* 108.7 (18.1) in the OBA group (difference 6.2; 95% confidence interval 0.4–12.0). We anticipate a similar effect size in our study. According to the literature,^16^ a change of 8.0 points in the QoR-15 score represents a clinically meaningful improvement or deterioration. A sample size of 90 patients per primary comparison group (choice *vs* randomisation) will provide ∼90% power to detect effect sizes as small as 0.5 (roughly equivalent to an 8-point difference on the QoR-15 scale) using a two-sample *t*-test (two-tailed, alpha=0.05). For downstream objectives, such as pairwise comparisons between the four groups (choice OFA, choice OBA, randomised OFA, randomised OBA), a sample size of 45 patients per group will provide 80% power to detect effect sizes as small as 0.6 (equivalent to a 9.6-point difference on the QoR-15 scale) using a two-sample *t*-test (two-tailed, alpha=0.05). These estimates are conservative simplifications of the proposed factorial model, which will have greater power by incorporating all data. As such, taking into account a 20–30% potential dropout for loss of postoperative assessment of QoR, we will include 240 patients in total.

## Statistical analysis

Before statistical modelling, patient characteristics and outcomes will be summarised by group using means (standard deviation) for continuous variables or frequencies (%) for categorical variables. Next, the primary analysis will use a factorial design framework to assess the primary outcome (QoR-15) using a linear model, including terms for group (choice *vs* no choice), anaesthetic type (OFA *vs* OBA), and their interaction, and preoperative factors (opioid use, history of chronic pain, history of drug abuse, history of PONV, history of previous abdominal surgery, age, sex) and surgery-related factors (duration of surgery and type of surgery). These adjustments will ensure that potential confounders are accounted for and will allow estimation of the overall effects of patient choice and anaesthetic type on QoR-15, while also enabling pairwise comparisons within and between the groups (e.g. comparing OFA *vs* OBA within both the choice and randomised groups).

To evaluate factors associated with anaesthesia preferences, univariable and multivariable logistic regression models will be used with patient choice (yes *vs* no) as the outcome and the aforementioned factors as covariates. Multicollinearity will be assessed using variance inflation factor statistics.

To further explore potential interactions between patient choice and anaesthesia type (OFA *vs* OBA), we will assess both additive and multiplicative interactions. For additive interactions, we will use linear regression models to test whether the combined effect of the two factors equals the sum of their individual effects, with interaction terms included in the model. For multiplicative interactions, we will evaluate whether the combined effect exceeds or is less than the product of the individual effects by log-transforming the outcome and fitting interaction terms in regression models. For binary outcomes, logistic regression will be used to assess interactions on the odds ratio scale. Additionally, we will calculate the relative excess risk attributable to interaction and the synergy index to quantify the magnitude and direction of interactions on the additive and multiplicative scales, respectively. If significant interactions are detected, stratified analyses will be conducted to examine the effect of one factor across levels of the other.

The primary analysis will follow an intention-to-treat (ITT) approach, including all participants regardless of anaesthesia protocol compliance. A per-protocol analysis will be performed for those with full adherence. An as-treated sensitivity analysis will also examine the impact of protocol adherence and actual treatment received. No interim analysis will be performed during the study.

## Missing values

Missing data will be addressed using appropriate statistical methods. Missing outcome data will be managed with multiple imputation if the missingness mechanism is likely to be missing at random (MAR). To ensure the robustness of the results, a sensitivity analysis will be conducted to evaluate the impact of different methods of handling missing data.

## Risk of bias

Efforts will be made to minimise the risk of bias in the study. Selection bias will be mitigated by randomisation for patients without a preference and by balanced recruitment with capped enrolment for the choice groups. Performance bias will be addressed by blinding postoperative care providers and evaluators and by standardising intraoperative and postoperative protocols. Detection bias will be reduced by ensuring that outcome assessors are blinded to group allocation. Attrition bias will be minimised by conservative sample size calculations that account for a 20–30% dropout rate. Reporting bias will be controlled by preregistering the study protocol and ensuring that all planned outcomes are fully reported.

## Patient and public involvement

Patient partners will collaborate with PERFECT study investigators to design a standardised, patient-friendly patient decision aid outlining the benefits and disadvantages of OBA and OFA. The aim is to create a clear, easily understandable document that respects health literacy principles, avoiding technical jargon to ensure patients can fully comprehend the information.

## Anticipated challenges

To address the challenge of protocol compliance with opioids, both ITT and per-protocol analyses will be used. Anaesthesiologists’ reasons behind protocol noncompliance or adherence may also be explored qualitatively. In the absence of a pilot study, patients’ preferences for OFA or OBA in the non-randomised group remain unknown, potentially leading to unequal group distribution. A capped enrolment strategy and an objective patient decision aid will help ensure balanced allocation across groups. In case of unexpected imbalance or discrepancy with previous observations, a mixed-method exploratory research will be considered to explain these observations and explore underlying reasons, informing practice and future research.

## Ethics statements

This study was approved by the Ethics Committee of UCLA (University of California Los Angeles) IRB-24-5789 on 24 February 2025 and was registered at Clinical Trial Registry under the number NCT06855641 (first posted: 2025-03-04). Before any changes, potential protocol amendments, including ancillary trials or changes to eligibility criteria, will be submitted to the UCLA ethics committee and communicated to relevant parties.

## Dissemination plans

Dissemination plans will include stakeholders and biostatisticians in the authors’ list, according to their study implication. Preliminary and final results will be presented at national and international conferences. Final results will be published in a peer-reviewed journal.

## Authors’ contributions

Study design and conception: all authors

Ethics committee process: NB, YG, AJ, MC

Methodology and statistical plan: TG

Patient decision aid redaction and evaluation: PF

Manuscript drafting: all authors

Approved the final version of this protocol: all authors

## Declaration of generative AI and AI-assisted technologies in the writing process

The authors used ChatGPT to improve English readability. After using this tool, the authors reviewed and edited the content as needed and take full responsibility for the content of the publication.

## Funding

This research received no specific grant from any funding agency in the public, commercial or not-for-profit sectors. The authors are solely responsible for the design, conduct, analysis, and reporting of this research. No external organisation or entity influenced any aspect of this study.

## Declarations of interest

PF is supported by the European Society of Anaesthesiology and Intensive Care (ESAIC) for the Pain and Opioids after Surgery (PANDOS) and the Euro-Periscope Research Groups (IDs ESAIC_GR_2021_PF, ESAIC_RG_PAND, and ESAIC_RG_EP), and received advisory board/speaker fees from Grunenthal, GE Healthcare, and Oncomfort.

YG receives speaker fees from GE Healthcare and received a personal financial support from the Phillip Foundation and Nîmes University Hospital for his UCLA research fellowship. MC and AJ are consultants for Edwards Lifesciences, Irvine, CA, USA

The other co-authors declare that they have no conflicts of interest.

## References

[1] Mattingly A.S., Chen M.M., Divi V., Holsinger F.C., Saraswathula A. (2023). Minimally invasive surgery in the United States, 2022: understanding its value using new datasets. J Surg Res.

[2] de Boer H.D., Detriche O., Forget P. (2017). Opioid-related side effects: postoperative ileus, urinary retention, nausea and vomiting, and shivering. A review of the literature. Best Pract Res Clin Anaesthesiol.

[3] Blum K.A., Liew L.Y., Dutia A.R. (2024). Opioid-free anesthesia: a practical guide for teaching and implementation. Minerva Anestesiol.

[4] da Silveira CAB., Rasador A.C.D., Medeiros H.J.S. (2024). Opioid-free anesthesia for minimally invasive abdominal surgery: a systematic review, meta-analysis, and trial sequential analysis. Can J Anaesth.

[5] Ao Y., Ma J., Zheng X., Zeng J., Wei K. (2025). Opioid-sparing anesthesia versus opioid-free anesthesia for the prevention of postoperative nausea and vomiting after laparoscopic bariatric surgery: a systematic review and network meta-analysis. Anesth Analg.

[6] Liu Y., Ma W., Zuo Y., Li Q. (2025). Opioid-free anaesthesia and postoperative quality of recovery: a systematic review and meta-analysis with trial sequential analysis. Anaesth Crit Care Pain Med.

[7] Pennington B.R.T., Politi M.C., Abdallah A.B. (2023). A survey of surgical patients’ perspectives and preferences towards general anesthesia techniques and shared-decision making. BMC Anesthesiol.

[8] Léger M., Perrault T., Pessiot-Royer S. (2024). Opioid-free anesthesia protocol on the early quality of recovery after major surgery (SOFA Trial): a randomized clinical trial. Anesthesiology.

[9] Chan A.W., Tetzlaff J.M., Gøtzsche P.C. (2013). SPIRIT 2013 explanation and elaboration: guidance for protocols of clinical trials. BMJ.

[10] Halvorsen S., Mehilli J., Cassese S. (2022). 2022 ESC Guidelines on cardiovascular assessment and management of patients undergoing non-cardiac surgery. Eur Heart J.

[11] Wasmann K.A., Wijsman P., van Dieren S., Bemelman W., Buskens C. (2019). Partially randomised patient preference trials as an alternative design to randomised controlled trials: systematic review and meta-analyses. BMJ Open.

[12] Stark P.A., Myles P.S., Burke J.A. (2013). Development and psychometric evaluation of a postoperative quality of recovery score: the QoR-15. Anesthesiology.

[13] Bauer M., Böhrer H., Aichele G., Bach A., Martin E. (2001). Measuring patient satisfaction with anaesthesia: perioperative questionnaire versus standardised face-to-face interview. Acta Anaesthesiol Scand.

[14] Herdman M., Gudex C., Lloyd A. (2011). Development and preliminary testing of the new five-level version of EQ-5D (EQ-5D-5L). Qual Life Res.

[15] Myles P.S., Shulman M.A., Heritier S. (2017). Validation of days at home as an outcome measure after surgery: a prospective cohort study in Australia. BMJ Open.

[16] Myles P.S., Myles D.B., Galagher W., Chew C., MacDonald N., Dennis A. (2016). Minimal clinically important difference for three quality of recovery scales. Anesthesiology.

