# Supplementary material for: Patient preference for intraoperative opioid use and early recovery after noncardiac surgery: protocol for a randomised factorial design trial of opioid-free versus opioid-based anaesthesia (the PERFECT trial)
Source: BJA Open. 2025 Jun 18;15:100420. doi: 10.1016/j.bjao.2025.100420 (PMC12219004; doi:10.1016/j.bjao.2025.100420)
Supplement: Multimedia component 2 [file mmc2.pdf]

Patient Preference for Intraoperative Opioid Use and Early Recovery Following Non-Cardiac Surgeries: A Randomized Factorial Design Trial of Opioid-Free versus Opioid-Based Anesthesia: The PERFECT Trial

Project Holder: Dr. Alexandre Joosten

City: Ronald Reagan Hospital, UCLA Health, Los Angeles, CA

1 Project coordinator

1.1.1 . Civility : M.

1.1.2 . Last Name : JOOSTEN

1.1.3 . First name : Alexandre

1.1.4 . City : Los Angeles, CA

1.1.5 . Email : ajoosten@mednet.ucla.edu

1.1.6 Position : MD, PhD

1.1.7 . Domain : Anesthesiology

2 Structures/Organizations

2.1 . Affiliated institution responsible for the budget from the ministry of health: XXX

2.2 . Financial manager : XXX

2.3 . Organization responsible for project management:

2.4 . Organization responsible for quality insurance : XXX

3 Research project

3.1.1. Project title: **Patient Preference for Intraoperative Opioid Use and Early Recovery Following Non-Cardiac Surgeries: A Randomized Factorial Design Trial of Opioid-Free versus Opioid-Based Anesthesia: The PERFECT randomized trial**

3.1 2. Acronym (w/o space) **PERFECT**

3.1.3. Field of study: Anesthesiology

3.1.4 . Free keywords: OFA, QoR-15, early recovery, opioid, postoperative pain trajectory, empowerment, shared decision-making.

3.1.5 . Ages of studied population: Age >18 years.

3.2 Rational (context and hypothesis):

Approximately 230 million major surgical procedures are conducted under anesthesia worldwide every year (1). Minimally invasive surgery volume has growing over the last years and became a gold standard approach for many surgical models to improve postoperative course. In parallel, new anesthesia trends in have been growing to offer better patients outcomes and postoperative comfort.
Opioid-Free Anesthesia (OFA) is an increasingly popular concept that allows the provider to administer safe general anesthesia without including opioids. Opioids are associated with well-recognized side effects such as nausea and vomiting, sedation, ileus, respiratory depression, increased acute and chronic postoperative pain, addiction, and potentials for misuse.

The OFA term encompasses a variety of techniques and substances, such as regional anesthesia, non-opioid medications, and a combination of analgesic modalities (2).
Some evidence has emerged that OFA offers several potential advantages for patients undergoing surgical procedures, including the reduction of postoperative nausea and vomiting (PONV) and postoperative pain (3,4). It also may contribute to an enhanced recovery with respect to a faster recovery (4,6,10).

In daily clinical practice, although providing necessary information is ethical and mandatory, patients rarely express preferences regarding the choice or combination of medications used in general anesthesia. Patients typically want to "go to sleep and wake up" without concern for the specifics. The decision about which type of general anesthetic to administer is typically made by the clinical team without patient engagement. Occasionally, they can make the choice of regional anesthesia versus general anesthesia, but even then, they typically rely on the expertise of the anesthesiologist. In a recent US survey, 43% of patients preferred a patient-led decision-making role, 28% preferred to share decision making with their clinical team and 29% prefer to have a physician-led process (9).

Involving patients in relevant shared decision-making is widely regarded as part of ideal patient care. This approach aims to bring together the patient's values and preferences with the physician's expertise to determine the best individualized care package.

In the context of the current opioid crisis in the USA, informing patients about their peroperative opioid exposure now seems more appropriate than ever. If clinically appropriate, offering an alternative OFA strategy ensures that patients can avoid a situation where they receive a medication they would wish to avoid. Indeed, while exact statistics are difficult to obtain, the perioperative period has been recognized as a gateway into opioid addiction (postoperative analgesia, postoperative pain, etc.).

Proposing OFA, and more generally allowing patients to choose any relevant components of their anesthesia, can provide a higher degree of patient autonomy and empowerment. In the context of recent literature, OFA research tends to currently focus on patient reported outcome measures (PROM) in the context of this novel anesthesia strategy. Currently conducted studies regarding OFA focus mainly on its quality of recovery (6, 7, 8,10), which is still in debate. But none have evaluated it in minimally invasive surgery context and neither the patient's choice regarding their own anesthetic plan. It is unknow that giving a patient an increased sense of autonomy, when applied to many safe anesthesia options, may affect their perception of and their actual recovery.

The aim of this study is to compare the effect of patient preference on intraoperative opioid use (opioid based anesthesia (OBA) vs OFA) on early postoperative quality of recovery following moderate risk laparoscopic/robotic abdominal surgery.
We hypothesize patients who choose their anesthesia strategy will have higher QoR-15 scores compare to patients whose anesthesia type is determined by random allocation.

(1) Weiser TG, Regenbogen SE, Thompson KD, Haynes AB, Lipsitz SR, Berry WR, et al. An estimation of the global volume of surgery: a modelling strategy based on available data. Lancet Lond Engl. 12 juill 2008;372(9633):139-44.

(2) Blum KA, Liew LY, Dutia AR, Aljohani DM, Bugada D, Forget P, Nesvadba DS. Opioid-free anesthesia: a practical guide for teaching and implementation. Minerva Anestesiol. 2024 Apr;90(4):300-310. doi: 10.23736/S0375-9393.23.17824-2. Epub 2024 Mar 14. PMID: 38482635.)

(3) Sin JCK, Tabah A, Campher MJJ, Laupland KB, Eley VA. The Effect of Dexmedetomidine on Postanesthesia Care Unit Discharge and Recovery: A Systematic Review and Meta-Analysis. Anesth Analg. 1 juin 2022;134(6):1229-44.

(4) Feenstra ML, Jansen S, Eshuis WJ, van Berge Henegouwen MI, Hollmann MW, Hermanides J. Opioid-free anesthesia: A systematic review and meta-analysis. J Clin Anesth. 2023 Nov;90:111215. doi: 10.1016/j.jclinane.2023.111215. Epub 2023 Jul 27. PMID: 37515877.

(5) Olausson A, Svensson CJ, Andréll P, Jildenstål P, Thörn SE, Wolf A. Total opioid-free general anaesthesia can improve postoperative outcomes after surgery, without evidence of adverse effects on patient safety and pain management: A systematic review and meta-analysis. Acta Anaesthesiol Scand. 2022 Feb;66(2):170-185. doi: 10.1111/aas.13994. Epub 2021 Nov 11. PMID: 34724195.

(6) Léger M, Perrault T, Pessiot-Royer S, Parot-Schinkel E, Costerousse F, Rineau E, Lasocki S. Opioid-free Anesthesia Protocol on the Early Quality of Recovery after Major Surgery (SOFA Trial): A Randomized Clinical Trial. Anesthesiology. 2024 Apr 1;140(4):679-689. doi: 10.1097/ALN.0000000000004840. PMID: 37976460.

(7) Zhou F, Cui Y, Cao L; Opioid-Free Anesthesia Working Group. The effect of opioid-free anaesthesia on the quality of recovery after endoscopic sinus surgery: A multicentre randomised controlled trial. Eur J Anaesthesiol. 2023 Aug 1;40(8):542-551. doi: 10.1097/EJA.0000000000001784. Epub 2023 Jun 29. PMID: 37377372.

(8) Feenstra ML, Jansen S, Eshuis WJ, van Berge Henegouwen MI, Hollmann MW, Hermanides J. Opioid-free anesthesia: A systematic review and meta-analysis. J Clin Anesth. 2023 Nov;90:111215. doi: 10.1016/j.jclinane.2023.111215. Epub 2023 Jul 27. PMID: 37515877.

(9) Pennington BRT, Politi MC, Abdallah AB, Janda AM, Eshun-Wilsonova I, deBourbon NG, Siderowf L, Klosterman H, Kheterpal S, Avidan MS. A survey of surgical patients' perspectives and preferences towards general anesthesia techniques and shared-

decision making. BMC Anesthesiol. 2023 Aug 17;23(1):277. doi: 10.1186/s12871-023-02219-5. PMID: 37592215; PMCID: PMC10433576.
(10): Liu Y, Ma W, Zuo Y, Li Q. Opioid-free anaesthesia and postoperative quality of recovery: a systematic review and meta-analysis with trial sequential analysis. Anaesth Crit Care Pain Med. 2024 Dec 11:101453. doi: 10.1016/j.accpm.2024.101453. Epub ahead of print. PMID: 39672303.

3.3 Originality and Innovative Aspects:

No large study has evaluated the impact of OFA versus OBA (standard anesthesia with opioids) on early postoperative recovery. Moreover, no studies have evaluated the impact of patient choice of these two strategies on outcome.

In this factorial trial, we will have the opportunity to study the interaction between the effect of technique and the influence of patient choice. This interaction may lead to a synergistic effect, because the effect of anesthesia technique (OFA vs. OBA) may be conditioned by patient preference (i.e. not only through an additive effect of satisfaction related to having choice, but also related to specific goals and the desire to avoid technique-specific side effects).

To provide appropriate information to patients, a patient partnership will be established with expert patients to draft the standardized information provided to patients (regarding the choice of strategy or not). The concept of shared decision-making is a central element of this study, in line with current recommendations on the importance of encouraging collaborative decision-making with surgical patients. Shared decision-making is rarely evaluated in the field of general anesthesia strategy and has never been explored in the context of OFA vs. OBA. In this study, the shared decision-making implementation will be aligned with the "SHARE approach" (6) encouraged and promoted by the Agency for Healthcare Research and Quality (AHRQ).

The current trends in anesthesia research and practice focus on Patient-Reported Outcome Measures and Experiences (PROMs & PREMs) to ensure an optimized perioperative course.
Recent international guidelines regarding Standardized Endpoints in Perioperative Medicine research have encouraged assessing postoperative recovery with validated tools in the field of perioperative studies, such as QoR-15 (1,2).

Patient satisfaction is typically assessed as a secondary outcome in the OFA literature using a simple Likert scale. In the current study, patient satisfaction will be assessed by a validated and recommended scale, the Bauer questionnaire (2,3).

Moreover, patient recovery is a key component of the value of care. The value of care in healthcare refers to the balance between the quality of services provided to patients and the costs associated with those services (4). It emphasizes achieving the best possible health outcomes for patients while efficiently utilizing resources. Value-based care aims to improve patient experiences and satisfaction, enhance health outcomes, and optimize costs, shifting the focus from quantity to the quality of care. Finally, assessing and improving patient-centered outcomes such as recovery are key responses to improving perioperative medicine delivery.

(1)Myles PS, Boney O, Botti M, Cyna AM, Gan TJ, Jensen MP, Kehlet H, Kurz A, De Oliveira GS Jr, Peyton P, Sessler DI, Tramèr MR, Wu CL; StEP–COMPAC Group; Myles P, Grocott M, Biccard B, Blazeby J, Boney O, Chan M, Diouf E, Fleisher L, Kalkman C, Kurz A, Moonesinghe R, Wijeysundera D. Systematic review and consensus definitions for the Standardised Endpoints in Perioperative Medicine (StEP) initiative: patient comfort. Br J Anaesth. 2018 Apr;120(4):705-711. doi: 10.1016/j.bja.2017.12.037. Epub 2018 Feb 2. PMID: 29576111.
(2) Moonesinghe SR, Jackson AIR, Boney O, Stevenson N, Chan MTV, Cook TM, Lane-Fall M, Kalkman C, Neuman MD, Nilsson U, Shulman M, Myles PS; Standardised Endpoints in Perioperative Medicine-Core Outcome Measures in Perioperative and Anaesthetic Care (StEP-COMPAC) Group. Systematic review and consensus definitions for the Standardised Endpoints in Perioperative Medicine initiative: patient-centred outcomes. Br J Anaesth. 2019 Nov;123(5):664-670. doi: 10.1016/j.bja.2019.07.020. Epub 2019 Sep 5. PMID: 31493848.
(3)Bauer M, Böhrer H, Aichele G, Bach A, Martin E. Measuring patient satisfaction with anaesthesia: Perioperative questionnaire versus standardised face-to-face interview. Acta Anaesthesiol Scand. 2001;45(1):65–72.
(4) Porter ME. What is value in health care? N Engl J Med. 23 déc 2010;363(26):2477-81.

<u>3.4 Expected benefit for patients or public health:</u>

The expected potential benefits are:

- For Patients: Experience a more comfortable postoperative course characterized by reduced PONV, postoperative pain, and enhanced postoperative recovery, contributing to a positive perioperative experience. Strengthening patient empowerment regarding their perioperative anesthesia through clear and honest information about the expected benefits and risks. Choosing their anesthesia strategy through the implementation of shared decision-making. Offering the possibility of opting for an opioid-free strategy in the context of the current opioid crisis.

- For Practitioners: Gain valuable feedback on patient recovery and satisfaction, aiding in the assessment of anesthesia strategies. Contribute to the implementation and learning of the shared decision-making concept.

- For learned societies: assist in editing international guidelines concerning the OFA delivery, which are currently lacking. This study aims to evaluate the impact of OFA on patient perspective. Incorporating patient preferences could provide a more realistic perspective on the effectiveness of OFA vs. OBA, potentially enhancing the external validity and applicability of the study's conclusions to everyday clinical practice.

- For Public Health and Payers: Evaluating anesthesia competencies, complementing the information provided by Surgical HCAHPS (Hospital Consumer Assessment of Healthcare Providers and Systems). Enhance the overall quality of care by leveraging patient-reported outcomes, supporting informed decision-making and resource allocation.

3.5 **Outcomes**

3.5.1: Primary outcome

Early postoperative quality of recovery on postoperative day 1 (POD#1) using the QoR-15 score (as a whole, and each item separately) (1) between patient who choose *vs* don't choose their anesthesia strategies.

The QoR-15 questionnaire will be filled by participants at hospital or completed by the investigator via phone call if patient will be already discharged. It consists of 15 questions covering five key dimensions of postoperative recovery: physical comfort, emotional state, psychological support, physical independence, pain management. It takes approximately **2.5 minutes** to complete. Each item is scored on a scale from 0 to 10, with higher scores indicating better recovery quality. The total score ranges from 0 to 150, providing a comprehensive assessment of the patient's recovery experience in the early postoperative period.

3.5.2: Secondary outcomes

Secondary outcomes are collected from patient's electronic medical record or participant phone call by investigators defined as follow

A. Early postoperative quality of recovery on POD#1, depending on OFA or OBA, whatever patient preferences: QoR-15 score, self-assessment at POD1.
B. PONV incidence, defined by the prevalence use (in %) of any antiemetic drug in the postoperative care unit (PACU), and from PACU discharge to home discharge.
C. Bauer questionnaire on POD1: anesthesia overall satisfaction and satisfaction rates by category prevalence (%) (2), self-assessment.
D. Early post-operative recovery on POD#2: QoR-15 score, self-assessment at POD2.
E. Opioid consumption: opioid consumption in morphine equivalent (in mg) from PACU to hospital discharge and from hospital discharge to POD30.
F. Bradycardia incidence: defined as the number of intraoperative episodes of heart rate < 40 with concomitant atropine administration (%).
G. Postoperative hypoxaemia incidence: defined as therapeutic oxygen supplementation to maintain SpO_2 > 95% from PACU arrival to POD#2.
H. Health quality of life on POD30: EuroQol 5 Dimension, five-level version (EQ-5D-5L) score with visual analogue scale (3)

(1): Stark PA, Myles PS, Burke JA. Development and psychometric evaluation of a postoperative quality of recovery score: the QoR-15. Anesthesiology. 2013 Jun;118(6):1332-40. doi: 10.1097/ALN.0b013e318289b84b. PMID: 23411725.

(2): *Bauer M, Böhrer H, Aichele G, Bach A, Martin E. Measuring patient satisfaction with anaesthesia: Perioperative questionnaire versus standardised face-to-face interview. Acta Anaesthesiol Scand. 2001;45(1):65–72.*

(3): Herdman M, Gudex C, Lloyd A, Janssen M, Kind P, Parkin D, Bonsel G, Badia X. Development and preliminary testing of the new five-level version of EQ-5D (EQ-5D-5L). Qual Life Res. 2011 Dec;20(10):1727-36. doi: 10.1007/s11136-011-9903-x. Epub 2011 Apr 9. PMID: 21479777; PMCID: PMC3220807.

Satisfaction with anaesthesia care

11.	How satisfied were you with the information you were given by the anaesthetist before the operation?			
	☐ Very satisfied	☐ Satisfied	☐ Dissatisfied	☐ Very dissatisfied
12.	How satisfied were you waking up from anaesthesia?			
	☐ Very satisfied	☐ Satisfied	☐ Dissatisfied	☐ Very dissatisfied
13.	How satisfied have you been with pain therapy after surgery?			
	☐ Very satisfied	☐ Satisfied	☐ Dissatisfied	☐ Very dissatisfied
14.	How satisfied were you with treatment of nausea and vomiting after the operation?			
	☐ Very satisfied	☐ Satisfied	☐ Dissatisfied	☐ Very dissatisfied
15.	How satisfied were you with the care provided by the department of anaesthesia in general?			
	☐ Very satisfied	☐ Satisfied	☐ Dissatisfied	☐ Very dissatisfied

Bauer questionnaire items 1

3.5.3 Exploratory outcomes:

Exploratory and safety outcomes are collected from patient's electronic medical record or phone call by investigators and defined as follow:

A. Intraoperative measures and anesthesia protocol adverse effects : anesthesia time (in min), prevalence (in %) of hypertension (SBP > 160mmHg), hypotension (MAP < 65mmHg), tachycardia (HR > 140bpm), anaphylactic reaction, use of amines, interruption of anesthesia drugs, blood loss (in mL) and blood transfusion (yes/no), prevalence (in %) of SPB < 90mmHg during PACU stay.
B. Time to awakening: time (in min) between the end of anesthesia gas administration and patient extubation.
C. Length of PACU & hospital Stays: the time of surgery to the time of discharge from PACU (in hours) and from hospital (in days).
D. Postoperative pain intensity (at rest) in PACU and from POD 1 to hospital discharge: assessed with a numerical rating scale (NRS) from 0 to 10 at rest.

E. Days at home for the 30 postoperative days: numbers of days alive and at home during the 30 days following surgery
F. Incidence of any postoperative surgical complication on POD30: according to the Clavien-Dindo classification.
G. Hospital readmission rate (%) on POD 30.

(1): Stark PA, Myles PS, Burke JA. Development and psychometric evaluation of a postoperative quality of recovery score: the QoR-15. Anesthesiology. 2013 Jun;118(6):1332-40. doi: 10.1097/ALN.0b013e318289b84b. PMID: 23411725.
(2) Bauer M, Böhrer H, Aichele G, Bach A, Martin E. Measuring patient satisfaction with anaesthesia: Perioperative questionnaire versus standardised face-to-face interview. Acta Anaesthesiol Scand. 2001;45(1):65–72.

<u>3.8 Study population:</u>

3.8.1 Main inclusion criteria:

- Age >18 years.
- Undergoing elective intermediate risk surgery under general anesthesia (robotic or laparoscopic assisted urological, gynecological or abdominal surgery).
- American Society Anesthesiologists grades I–IV.
- English-speaking.
- Informed consent signed.

3.8.2 Main exclusion criteria:

- Pregnancy or lactation.
- History of mental disorders.
- Contraindications to study drug (lidocaine, magnesium, dexmedetomidine, ketamine)
- Patient is participating in another interventional trial
- Patient is under judicial protection or is an adult under guardianship
- Patients who have a diagnosis of chronic pain
- Patients who are prescribed opioids

4 Methodology and inclusions

<u>4.1 Study design:</u>

The PERFECT trial is a prospective interventional pragmatic monocenter controlled partially randomized trial of superiority, conducted at Ronald Reagan and Santa Monica UCLA Medical Center, Los Angeles, USA.
This tertiary teaching hospital performs sufficiently minimally invasive abdominal surgeries each year. Patient enrollment plan is between March 2025 and March 2026, within a 12 months period recruitment.

Patient selection and consent:

According to inclusion criteria, PERFECT trial investigators will identify eligible participants the day before surgery: participants will receive a phone call and email of the consent form for review providing standardized information about protocol, OFA and OBA. Written consent will be obtained on the day of surgery, prior to anesthesia.

Allocation and blinding

Factorial design details
To assess the effects of OFA and OBA on early postoperative recovery, the factorial design for this study will include two key factors: patient choice and anesthetic type.
The patient choice factor will assess whether patients choose their anesthesia strategy, according to a patient-led decision or a shared-decision with clinicians, or will be randomized if denied making an anesthetic choice.
The anesthetic type factor will evaluate the impact of OFA versus OBA.
This will create a 2x2 factorial design, resulting in four groups:

- **Group 1**: "OFA-C" OFA - Patient Choice = Patients choosing to receive opioid-free anesthesia. (Patient-led decision or shared-decision).
- **Group 2**: "OBA-C" OBA - Patient Choice = Patients choosing to receive traditional opioid-based anesthesia. ((Patient-led decision or shared-decision).
- **Group 3**: "OFA-R" OFA – Randomization = Patients with no preference, randomized to receive opioid-free anesthesia.
- **Group 4**: "OBA-R" OBA – Randomization = Patients with no preference, randomized to receive traditional opioid-based anesthesia

This design allows us to test the main effects of patient choice and anesthesia type and to explore potential interaction effects between these two factors.

To asses how a patient choice impacts outcomes, this study will be partially randomized: Participants denied making an anesthetic choice will be randomized whereas participants making a choice won't be.
Randomized Patient preference Trials (RPPT) seems to be a reliable alternative for RCTs (1), especially in trials using patient-centered outcomes. In case patients' preference can be assumed, RPPT enables faster inclusion of a more representative population improving external validity without compromising internal validity.
The study flow diagrams are summarized in figure 1 and 2:

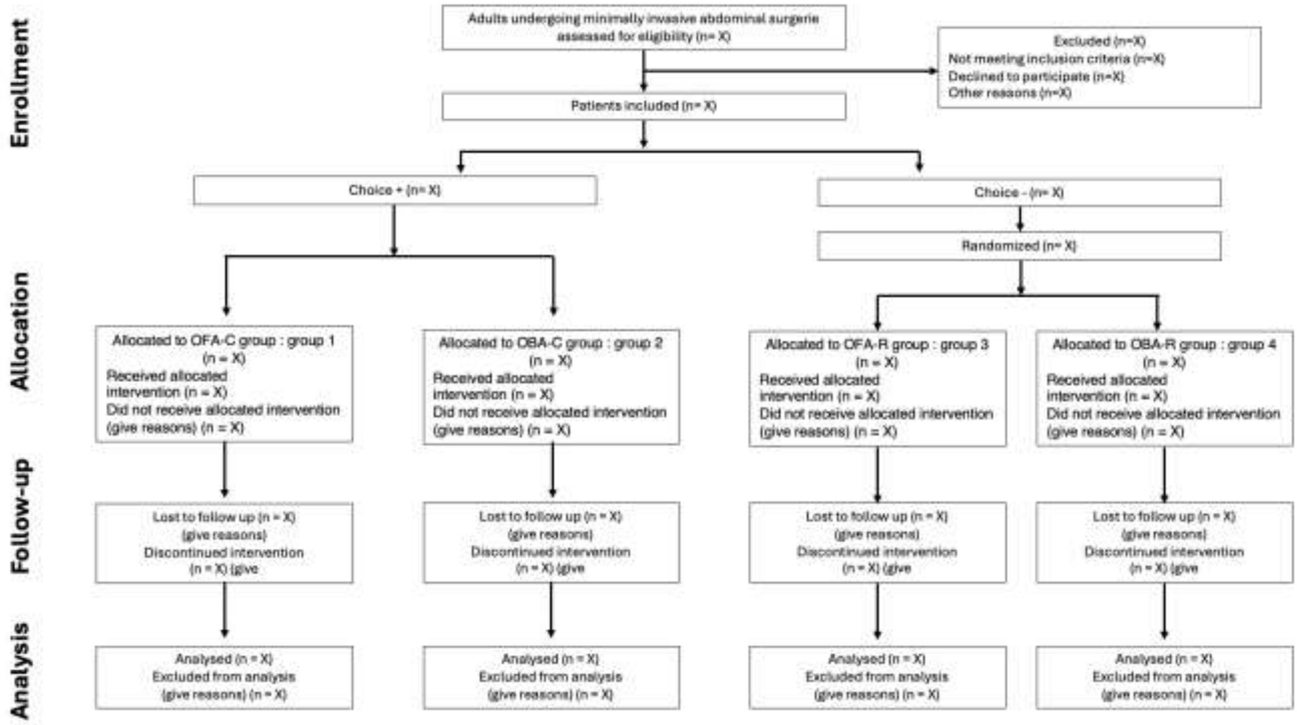

Fig.2 PERFECT Study flow chart

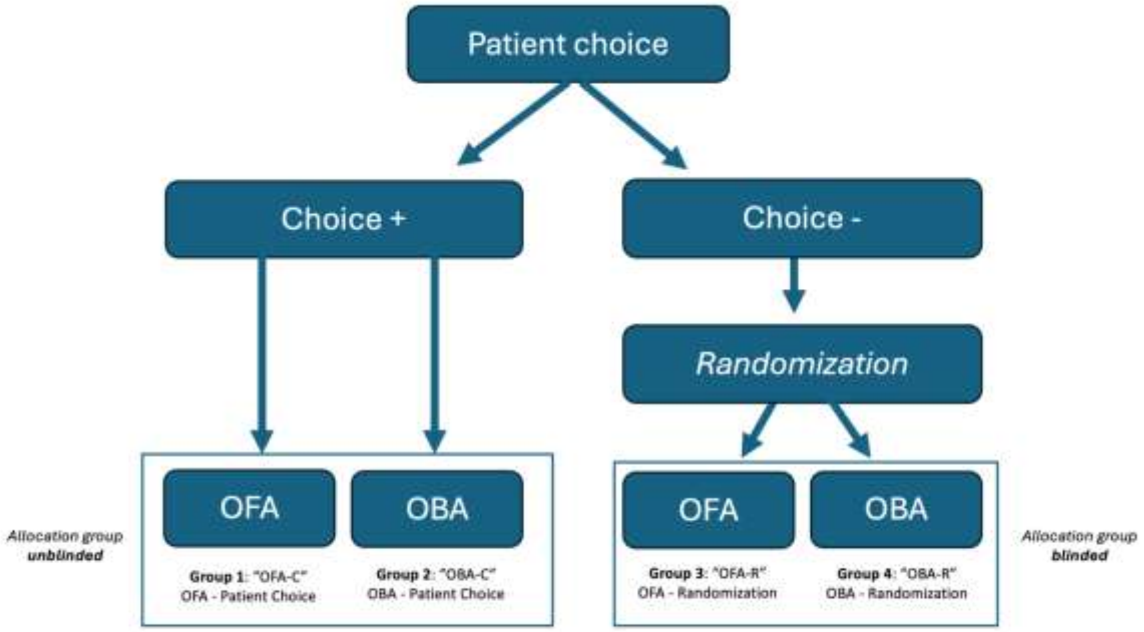

Fig.2 Study design

Patient Distribution:

To ensure balanced group sizes, different approaches will be employed for the choice and no-choice groups:

- For the randomized groups 3 & 4 (OFA-R and OBA-R), we will use a 1:1 allocation ratio to ensure equal distribution between the randomized groups. A permuted block design (block size of 6) will be used to ensure balanced group sizes while incorporating randomization within each block. A randomization list using R V4.1.0 ('block rand' package) will determine the sequence of assignments, ensuring balance and preventing predictability. This method will ensure that the groups remain balanced throughout the study while reducing the risk of allocation bias. The randomization list will be generated using R V 4.1.0 (www.r-project.org Vienna, AU) using the 'blockrand' package (Schulz, K. and Grimes, D. (2002): Unequal group sizes in randomized trials: guarding against guessing, The Lancet, 359, pp 966–970.)"
- For the choice groups 1 & 2, participants won't be randomized. The target will be 50 participants per group. However, because group assignment is based on patient preferences rather than randomization, achieving an exact 1:1 ratio may not be possible. To address this, a capped enrollment strategy will be employed. This approach ensures that enrollment into each choice group is monitored, and recruitment will stop for a group once its target size has been reached. This method minimizes imbalance while respecting patient autonomy in selecting their preferred anesthesia strategy.

Blind status:
Blinding will be partially maintained in this study. The anesthesia team performing the general anesthesia will be unblinded on the allocation group, like participants in groups 1 & 2 (OFA-C & OBA-C). Anesthesia techniques not allowed anesthesia providers being blinded, according to different anesthesia drugs regimens. Participants in groups 3 & 4 (OFA-R & OBA-R), surgeons, postoperative care providers, evaluators and statistician will be blinded on the allocation group.
To maintain the integrity of blinding, separate personnel will handle intraoperative (unblinded) and postoperative (blinded) care and evaluation. Additionally, postoperative recovery protocols will be standardized across all groups to minimize potential bias.

(1): Wasmann KA, Wijsman P, van Dieren S, Bemelman W, Buskens C. Partially randomised patient preference trials as an alternative design to randomised controlled trials: systematic review and meta-analyses. BMJ Open. 2019 Oct 16;9(10):e031151. doi: 10.1136/bmjopen-2019-031151. PMID: 31619428; PMCID: PMC6797441.

<u>4.2: Description of the Experimental Design and Study Progression:</u>

The schedule of patient enrolment, study interventions and outcome assessment is in accordance with the SPIRIT statement (table 1). The total participant participation duration will be 30 days.

<u>Before surgery:</u>
Patients will be informed of the study during the pre-operative anesthesia consultation before surgery. Patients meeting inclusion criteria will be included in the trial after signing consent.

The day before surgery, participants will receive a phone call from PERFECT investigators to explain the study and provide standardized information about the benefits and drawbacks of OBA and OFA, as well as their options (choosing or randomization). Participants will also receive an email of the consent form for review providing standardized information.
This information will follow a written template created by the study team and patient partners. There is no specific time limit for this discussion; it will conclude once the participant has understood the key points of the protocol, including the OBA and OFA strategies.
We will assess participants' understanding through open-ended questions that require them to explain key elements of the study in their own words, including the objectives, procedures, risks, and benefits. Clarifications will be provided as needed until comprehension is confirmed.
On the day of surgery, in the preoperative area, participants will decide whether to join the study and, if so, whether to choose their anesthesia strategy or opt for randomization. Written consent will be obtained on the day of surgery, prior to anesthesia.
Family members may assist in the decision if requested. Participants will also give feedback on the anesthesia choice process.

Preoperative assessment at baseline:

- Demographic data and medical history.
- Preoperative morphine requirement and pain score at rest.
- Factors associated with the choice of anesthesia strategy: age, sex, history of opioid use or addiction, smoking status, substance or drug abuse, history of chronic pain, prior PONV or excessive sedation, previous abdominal surgeries, and the type of scheduled surgery

Standardized general anesthesia protocol: all groups

- Premedication allowed
- Anesthetic monitoring modalities including pulse oximetry, electrocardiography, noninvasive blood pressure and/or invasive monitoring when indicated, body temperature, muscle relaxation (train-of-four stimulation) and depth of anesthesia monitoring.
- Anesthesia induction at the anesthesiologist discretion.
- Anesthesia maintenance with halogenated anesthesia (Sevoflurane or desflurane) and neuromuscular blocking agents at the anesthesiologist’s discretion in both groups.
- Intraoperative hemodynamic maintenance with a range of IV vasoconstrictive drugs with the goal of MAP > 65mmHg.
- The use of regional anesthesia (parietal plane block), without opioids adjuvant in the OFA group.
- Wound infiltration with subcutaneous Bupivacaine
- Postoperative analgesia with a multimodal analgesia strategy including IV Acetaminophen, +/- IV Non-steroidal anti-inflammatory drug, according to potential patient contraindications and currents guidelines.
- PONV prevention with IV dexamethasone 4-10mg and IV Ondansetron 4mg prior to general anesthesia end time. 0.625mg of IV Droperidol, will be administered based on the Apfel score to address the risk of PONV.

Participants from OBA group:
The protocol includes opioids (maximum of fentanyl 100 mcg) for induction and anesthesia maintenance, which additional boluses (up to 50 mcg per bolus) at the anesthesiologists discretion. IV Lidocaine bolus with induction (up to 1mg/kg) is permitted as this is considered routine practice for induction.
Compliance with the protocol will be no IV ketamine, no IV magnesium and no IV dexmedetomidine will be administered.

Participants from OFA group:
The protocol includes no opioid drugs and at least 2 infusions among: IV ketamine, IV lidocaine, IV dexmedetomidine or IV magnesium:

- IV magnesium: at induction (40 mg/kg). Maximum dose: 3g
- IV lidocaine: 1.5-mg/ kg bolus dose in 10 min at induction followed by 1.5-mg/kg/h continuous infusion, stopped 30-60 minutes prior to end of surgery. If local anesthesia is used by surgical team, then no loading dose
- IV dexmedetomidine: 0.5µg/kg in 10min at induction, followed by an hourly infusion equal to 50% of the loading dose.
- IV ketamine: 0.5 mg/kg bolus dose at induction followed by a 0.2 mg/kg/h continuous infusion, stopped 30min before end of surgery.

Protocol compliance will be: at least two of the previous drugs are used and no intraoperative administrated opioids. Dosages are based on current literature for recommended dosages as well as routine standards for drug dose administration at UCLA (both Ronald Reagan UCLA Medical Center and UCLA Santa Monica Medical Center in Los Angeles.

Standardized postoperative protocol: all groups
In PACU, acute pain is treated using IV opioid titration until pain relief is achieved (NRS <3/10).
PONV: Standardized nausea and vomiting medications will be ordered for all patients
Multimodal postoperative analgesia is prescribed to all patient, according to potential patient contraindications, including a rescue opioid treatment.

OFA and OBA anesthesia will be provided by trained anesthesiologist from UCLA hospital. Compliance will be recorded by investigators from participant's electronic medical record and considered achieved regardless of the specific doses used, and protocol adherence will be validated even if the infusion is stopped early or if the initial bolus is not administered.

On POD1, POD2 and until hospital discharge:
The patient will self-assess:

- POD1 and POD2: The QoR-15 score (primary outcome). In case of difficulties reading and responding to the questionnaire, the patient may be assisted in completing it by one of the study evaluators, nurse or a relative.
- POD1: Patient satisfaction related to anesthesia.
- Postoperative pain intensity (at rest) from POD1 to hospital discharge.
- Post operative morphine consumption will be collected from the computerized patient record from POD1 to hospital discharge.

- Length of PACU & hospital stays will be collected from the computerized patient record at hospital discharge.

If the patient has already been discharged from the hospital, the completion of the questionnaires, the evaluation of pain scores, opioid requirement and PONV will be conducted via a phone call.

<u>On POD30:</u>

- Postoperative Complications: from the computerized patient record.
- Days at home for the 30 postoperative days: from the computerized patient record or phone call if unavailable.
- Hospital readmission rate: from the computerized patient record
- Opioid Consumption, by phone call.
- Health quality of life: EQ-ED-5L VAS score, by phone call.

Table 1: Schedule of enrolment, study interventions, and outcome assessments.

	STUDY PERIOD								
	Enrolment	Allocation	Post-allocation					Close-out	Follow-up
TIMEPOINT**	*Preop visit*	Before GA	*Intraoperatively*	*PACU*	*POD 1*	*POD 2*	*POD X*	*Hospital discharge*	*POD30*
**Patient enrolment**									
Eligibility criteria	X								
Written informed consent	X								
Demographic data	X								
Baseline characteristics	X								
Randomization/ Allocation		X							
Factors associated with choice		X	X						
**Study interventions:**									
OFA-OBA-no preference choice		X							
OFA: opioid-free-anesthesia			X						
OBA: opioid-based-anesthesia			X						
**Outcome assessment:**									
Bradycardia			X						
Surgical and anesthesia characteristics			X						
Intraoperative data and complications			X	X					
Postoperative hypoxemia				X	X	X			
QOR-15					←—	—→			
PONV				←—	—	—	—	—→	
Patient satisfaction					X				
Opioid consumption	X			←—	—	—	—	—	—→
Postoperative pain scores				←—	—	—	—	—→	
Length of PACU stay				X					
Length of hospital stay								X	
DAH30									X
EQ-5D-5L									X
Postoperative complications									X
Readmission rate									X

According to SPIRIT statement of defining standard protocol items for clinical trials.

DAH30: Days at home 30; EQ-5D-5L: EuroQol 5 Dimension, five-level version with visual analogue scale; GA: general anesthesia; PACU: postoperative care unit; POD: postoperative day; PONV: postoperative nausea and vomiting; QoR-15: quality of recovery 15.

4.3. Inclusions/Enrollment:

4.3.1 Does the project include enrollment or participation? **Yes**

4.3.2 Duration of participation (duration): **30 days**

4.3.3 Duration of participation (unit of duration): **Day(s)**

4.3.4 Anticipated duration of recruitment (DUR) (in month): **12**

4.5.5 Total number of scheduled participants to be recruited or observations to be collected (NP): **240**

4.4 Sample size justification and statistical analysis:

In a previous study conducted in 2021 (1) on adult perioperative anesthesia outcomes in France (N=135 patients), using a similar anesthesia protocol and inclusion criteria, the mean Quality of Recovery-15 (QoR-15) score at 24 hours was 114.9 ± 15.2 in the opioid-free anesthesia (OFA) group versus 108.7 ± 18.1 in the standard opioid-based anesthesia (OBA) group (difference: 6.2; 95% CI: 0.4 to 12.0; P = 0.026) . We anticipate a similar effect size in our proposed study. According to the literature, a change of 8.0 points in the QoR-15 score represents a clinically meaningful improvement or deterioration (2). A sample size of 90 patients per primary comparison group (choice vs. randomization) will provide approximately 90% power to detect effect sizes as small as 0.5 (roughly equivalent to an 8-point difference on the QoR-15 scale) using a two- sample t-test (two-tailed, alpha=0.05). For downstream objectives, such as pairwise comparisons between the four groups (choice OFA, choice OBA, randomized OFA, randomized OBA), a sample size of 45 patients per group will provide 80% power to detect effect sizes as small as 0.6 (equivalent to a 9.6-point difference on the QoR-15 scale) using a two-sample t- test (two-tailed, alpha=0.05). These estimates are conservative simplifications of the proposed factorial model, which will have greater power by incorporating all data. As such, taking into account a 20-30% potential drop out for loss of postoperative assessment of quality of recovery, we will include 240 patients in total.

Prior to statistical modeling, patient characteristics and outcomes will be summarized by group using means (SD) for continuous variables or frequencies (%) for categorical variables. Next, the primary analysis will employ a factorial design framework to assess the primary outcome (QoR-15) using a linear model, including terms for group (choice vs. no choice), anesthetic type (OFA vs. OBA), and their interaction, as well as preoperative factors (opioid use, history of chronic pain, history of drug abuse, history of post operative nausea and vomiting (PONV), history of previous abdominal surgery, age, sex) and surgery- related factors (duration of surgery and type of surgery). These adjustments will ensure that potential confounders are accounted for and will allow estimation of the overall effects of patient choice and anesthetic type on QoR-15, while also enabling pairwise comparisons within and

between the groups (e.g., comparing OFA vs. OBA within both the choice and randomized groups).
To address the secondary objective of evaluating factors associated with the choice of anesthesia strategy (OFA vs. OBA), relevant patient characteristics will be collected to explore patterns and associations in their decision-making. These factors will include age, sex, history of opioid use or addiction, smoking status, history of substance or drug abuse, chronic pain history, prior postoperative nausea and vomiting (PONV) or excessive sedation, previous major surgeries, and the type of scheduled major surgery. For this objective, univariable and multivariable logistic regression models will be employed with patient choice (yes vs. no) as the outcome and the aforementioned factors as covariates. Multicollinearity will be assessed using variance inflation factor (VIF) statistics.
Compliance with the protocols will be defined as follows: the OFA protocol will be considered complete if at least two of the following drugs are used—ketamine, lidocaine, dexmedetomidine and magnesium sulfate—and if no intraoperative opioids are administered. The standard OBA protocol will be considered complete if IV lidocaine, dexmedetomidine or magnesium sulfate are not used intraoperatively (ketamine is allowed). Compliance will be considered achieved regardless of the specific doses used, and protocol adherence will be validated even if the infusion is stopped early or if the initial bolus is not administered.
No interim analysis will be performed during the study to maintain the integrity of the primary outcome assessment and to avoid inflating the risk of Type I error. This approach is feasible given the manageable sample size of 240 patients and the study's relatively short duration of 12 months.

Missing values
Missing data will be addressed using appropriate statistical methods. Missing outcome data will be managed with multiple imputation if the missingness mechanism is likely to be Missing at Random (MAR). To ensure the robustness of the results, a sensitivity analysis will be conducted to evaluate the impact of different methods of handling missing data.

Risk of bias
Efforts will be made to minimize the risk of bias in the study. Selection bias will be mitigated by randomization for patients without a preference and by balanced recruitment with capped enrollment for the choice groups. Performance bias will be addressed by blinding postoperative care providers and evaluators and by standardizing intraoperative and postoperative protocols. Detection bias will be reduced by ensuring that outcome assessors are blinded to group allocation. Attrition bias will be minimized by conservative sample size calculations that account for a 10% dropout rate. Reporting bias will be controlled by pre-registering the study protocol and ensuring that all planned outcomes are fully reported.

4.4 Data collection and monitoring:

Data will be collected into a CRF by investigators blinded to the allocation group.
All data will be securely stored on a password-protected server hosted at UCLA, in compliance with institutional data security policies and regulatory requirements. Access to the data will be restricted to authorized study personnel only. Each participant will be assigned a unique study identification number to ensure confidentiality and anonymity.

No data monitoring committee will be involved, due to the low risk intervention and single center design.

REFERENCES

(1) Léger M, Perrault T, Pessiot-Royer S, Parot-Schinkel E, Costerousse F, Rineau E, Lasocki S. Opioid-free Anesthesia Protocol on the Early Quality of Recovery after Major Surgery (SOFA Trial): A Randomized Clinical Trial. Anesthesiology. 2024 Apr 1;140(4):679-689. doi: 10.1097/ALN.0000000000004840. PMID: 37976460.

(2) Myles PS, Myles DB, Galagher W, Chew C, MacDonald N, Dennis A. Minimal Clinically Important Difference for Three Quality of Recovery Scales. Anesthesiology. 2016 Jul;125(1):39-45. doi: 10.1097/ALN.0000000000001158. PMID: 27159009.

:
