# Supplementary material for: Patient preference for intraoperative opioid use and early recovery after noncardiac surgery: protocol for a randomised factorial design trial of opioid-free versus opioid-based anaesthesia (the PERFECT trial)
Source: BJA Open. 2025 Jun 18;15:100420. doi: 10.1016/j.bjao.2025.100420 (PMC12219004; doi:10.1016/j.bjao.2025.100420)
Supplement: Multimedia component 3 [file mmc3.pdf]

UNIVERSITY OF CALIFORNIA LOS ANGELES
CONSENT TO PARTICIPATE IN RESEARCH

Patient Preference for Intraoperative Opioid Use and Early Recovery Following Non-Cardiac Surgeries: A Randomized Factorial Design Trial of Opioid-Free versus Opioid-Based Anesthesia

The "PERFECT" Trial

INTRODUCTION

Drs. Alexandre Joosten and associates from the Department of Anesthesiology and Perioperative Medicine at the University of California, Los Angeles are conducting a research study. This study is funded by the Department of Anesthesiology and Perioperative Medicine at the University of California.

KEY INFORMATION

The following provides a short summary of this study to help you decide on whether or not to participate.

WHY AM I BEING INVITED TO TAKE PART IN A RESEARCH STUDY?

We invite you to take part in a research study because you are scheduled for intermediate to major non-ambulatory (inpatient) surgery estimated to last at least 60 min. The results of this study may help anesthesia providers around the world make better decisions for managing and improving patients' pain after surgery.

WHAT SHOULD I KNOW ABOUT THIS RESEARCH STUDY?

- Someone will explain this research study to you.
- Whether or not you take part is up to you.
- You can choose not to take part.
- You can agree to take part and later change your mind.
- Your decision will not be held against you.
- You can ask all the questions you want before you decide.
- You can discuss this study with friends and family.
- You can also discuss it with your health care doctor or request a second opinion.

WHY IS THIS RESEARCH BEING DONE?

Patients undergoing general anesthesia typically receive IV opioids, such as fentanyl, to maintain anesthesia stability and ensure comfort during surgery and in the immediate recovery phase, which is typically 1-4 hours for most patients. However, opioids are linked to well-known side effects, such as nausea, vomiting, sedation, respiratory depression, increased postoperative pain, and the risk of addiction or misuse. Given the current opioid

crisis in the USA, it is important to inform patients about their opioid exposure before, during, and after surgery.

An alternative approach, Opioid-Free Anesthesia (OFA), has been developed to avoid the use of intraoperative opioids (opioids during surgery). OFA offers several benefits, including reduced postoperative nausea, vomiting, and pain, and may promote a faster recovery, allowing patients to resume daily activities more quickly.

Despite these benefits, the choice between OFA and opioid-based anesthesia (OBA) is rarely discussed in routine practice. We believe that allowing patients to choose their anesthesia strategy has the potential to impact the quality of early recovery after surgery. Offering this choice promotes shared decision-making and empowers patients.

The goal of this study is to understand how your choice of anesthesia affects your recovery after surgery. Specifically, we want to see if choosing between opioid-free anesthesia (OFA) or opioid-based anesthesia (OBA) makes a difference in how well you recover after your surgery.

In this study, there will be four groups. If you have a preference for which type of anesthesia you want, you will choose between OFA or OBA after getting all the necessary information. If you don’t have a preference, you will be randomly assigned to either OFA or OBA.

The groups are:

1. People who choose OFA.
2. People who choose OBA.
3. People who are randomly assigned to receive OFA
4. People who are randomly assigned to receive OBA

The intervention groups consist of patients who choose to receive either opioid-free anesthesia (OFA) or opioid-based anesthesia (OBA) after being informed. These include patients choosing OFA and those choosing OBA. The control groups consist of patients who express no preference and are randomly assigned to receive either OFA or OBA. These include patients randomly assigned to OFA and those randomly assigned to OBA.

If you are in the **OFA group**, you will receive other medications instead of opioids to ensure your anesthesia is safe and works smoothly during surgery.

If you are in the **OBA group**, you will receive opioids as part of the standard care.
In both groups, you will be fully asleep during the surgery. The quality of anesthesia and pain control will be the same for both groups.

We expect that your participation in this research study will last for the duration of your surgery, with follow-up data collected up to 30 days after your procedure. More detailed information about the study procedures can be found under the section "WHAT WILL HAPPEN IF I TAKE PART IN THIS STUDY?"

WHAT ARE THE RISKS I SHOULD EXPECT?

There is a rare risk of breach of confidentiality related to the study data. Although very uncommon, more detailed information about the risks of this study can be found under

"WHAT KINDS OF RISKS OR DISCOMFORTS COULD I EXPECT? (Detailed Description)"

Both opioid-free anesthesia (OFA) and opioid-based anesthesia (OBA) have some risks. These risks have been well studied in medical research and are known to anesthesiologists.

The FDA has approved the use of all the drugs you will be exposed to in this study. As with any anesthetic received, heart rate, oxygen levels, and blood pressure will be closely checked during anesthesia and in the recovery room, in order to make adjustments and interventions as necessary.

Nausea and Vomiting:
With OBA, you may feel more nausea or vomiting after the surgery. These symptoms are linked to the opioids and anesthesia gases you will receive during the procedure. OFA, which does not use opioids, reduces the likelihood of nausea and vomiting after surgery. Regardless of which type of anesthetic is received, nausea and vomiting will be treated. Additionally, because the risk of nausea and vomiting cannot be eliminated, no matter which anesthesia is used (OFA or OBA), your anesthesiologist will give you preventive medications during the procedure to further reduce the risk of nausea and vomiting.

Pain Control:
Your pain after the surgery will not be different depending on the type of anesthesia used. Both OFA and OBA work equally well to manage pain. During the operation and in the postoperative recovery unit, your anesthesiologist will give you medications for pain management. After that time, your surgical team will provide their routine pain medications if necessary. We are only studying the pain medications used during your surgery and in the immediate recovery area.

Blood Pressure and Heart Rate:
During general anesthesia, it is extremely common for your blood pressure and heart rate to fluctuate. This can happen to every patient, regardless of their age, gender, type of surgery, or type of anesthetic used. These changes are routinely monitored by anesthesiologists, all patients receiving a general anesthetic are under continuous monitoring, and any intervention necessary will be given as is done for every anesthetic. Studies have shown that there is not a significant difference in how OFA and OBA affect blood pressure and heart rate.

ARE THERE ANY BENEFITS IF I PARTICIPATE?

We cannot promise any benefits to you or others from your participation in this research. However, possible benefits for the intervention group include the potential for an improved early quality of recovery after surgery, allowing you to return to your daily activities more quickly.

The knowledge gained from this study may provide a better understanding of the factors associated with the choice of anesthesia strategy and insights into early postoperative recovery and patient satisfaction with OFA.

WHAT OTHER CHOICES DO I HAVE IF I DON'T WANT TO PARTICIPATE?

Participation in research is completely voluntary. You can decide to participate or not to participate. Your alternative to participating in this research study is to not participate. Use of the OFA or choosing between OBA or OFA are not considered part of standard care at UCLA. If you do not participate in the study, you would receive standard care with opioid based anesthesia as described for the OBA group.

HOW MANY PEOPLE WILL TAKE PART IN THIS STUDY?

Approximately 240 people will take part in this study at UCLA.

WHAT WILL HAPPEN IF I TAKE PART IN THIS STUDY?

Before you begin the study:
The following definitions may help you understand how this research study is designed.

This study is a research project led by the researchers, aiming to test if one treatment is better than another. It will be done at Ronald Reagan UCLA Medical Center and UCLA Santa Monica Medical Center in Los Angeles. The study will compare different treatments, and some participants will be randomly assigned to different groups. Patients who are randomly assigned to different groups, as well as the researchers and the statistician, will not know which treatment the participants are receiving.

This study uses a special method to organize participants into different groups. If you have a preference for which type of anesthesia you want, you will be able to choose between two options: Opioid-Free Anesthesia (OFA) or Opioid-Based Anesthesia (OBA). If you don't have a preference, you will be randomly assigned to one of the two anesthesia options.

The anesthesiologist will explain the risks and benefits of both OFA and OBA, taking into account your medical history and the type of surgery you're having. If you don't meet the study's criteria, you won't be able to participate.
This approach allows us to see how your personal choice affects recovery and also helps us compare how each anesthesia type affects recovery. The random assignment will be done using a computer program to ensure fairness.

If you decide not to choose your anesthesia type, you will be randomly placed in either the OFA or OBA group. If you do choose, you will be assigned to the group based on your decision.

Your surgery and anesthesia will not be affected by which group you're in, and the anesthesia will take the same amount of time for everyone. The anesthesiologist will know which group you're in when you give consent, and if you choose, you'll also know which group you're assigned to.

During the study:

If you agree to participate in this study and after signing the consent form, the following things will occur:

- If you are in the interventional group: you will choose your anesthesia strategy, either OFA or OBA.
- If you are in the control group: you will be randomly assigned to OFA or OBA and will be blinded to your group allocation.

In the OBA group: the following things will occur:

- There is no change in the way the anesthesia team manages anesthesia during surgery when compared to the standard care already practiced at this hospital. The intraoperative opioid administration will therefore be exactly what is done during a standard surgical case in this hospital.

In the OFA group: the following things will occur:

- No opioids will be used, and at least two infusions of alternative pain management therapies will be administered for anesthesia induction and maintenance: IV ketamine, IV lidocaine, IV dexmedetomidine, or IV magnesium.

For both groups:

- Data will be collected from your medical record during your hospital stay and at postoperative day 30 following your surgery.
- If you are discharged from the hospital on day 1 or day 2, the study outcomes will be collected via phone call.
- You will complete a quality of recovery questionnaire, **the QoR-15**, on postoperative days 1 and 2. This questionnaire consists of 15 questions in English and takes approximately 2.5 minutes to complete. If you have difficulty reading or understanding the questions, a relative, a nurse, or a member of the research team can assist you in completing it.
- You will receive a phone call on postoperative day 30 to complete the EQ-5D-5L quality of life questionnaire. This questionnaire consists of six questions and takes less than five minutes to complete. As with your responses to other questionnaires, all your answers will remain anonymous and will not be shared with your healthcare providers

- A blinded research personnel (unaware of the group assignment) will collect and record the following perioperative (before, during, and in the immediate recovery phase after surgery) data while you are in the study, up to 30 days after surgery:
  - Date of birth, age, sex, education level, ethnicity, height, weight, medications, preoperative condition, history of opioid use or addiction, smoking status, substance or drug abuse, chronic pain, prior postoperative nausea and vomiting, prior excessive postoperative pain.
  - Surgical procedure data, including type of surgery, vital signs, laboratory, volume of products given, medications, and other clinical information.
  - Outcome data: length of stay, complications.

- Follow-up data includes:
  - Day 0 (Day of surgery): All data from during surgery, opioid requirements before surgery and factors associated with choice.
  - Postoperative (After Surgery) Day 1: QoR-15 questionnaire, opioid requirement, patient satisfaction, pain intensity, postoperative nausea.
  - Postoperative (After Surgery) Day 2: QoR-15 questionnaire, opioid requirement, pain intensity, postoperative nausea.
  - From postoperative (After Surgery) Day 2 to hospital discharge: opioid requirement, pain intensity, postoperative nausea.
  - At hospital discharge: opioid requirement, pain intensity, nausea in the immediate recovery phase after surgery, hospital length of stay.
  - Postoperative (After Surgery) Day 30: surgical complications, readmission rate, opioid requirement, EQ-5D-5L score and days at home at 30 days after surgery.

Here is the table summarizing the information collected at the different stages of the PERFECT study:

	STUDY PERIOD								
	Enrolment	Allocation	Post-allocation					Close-out	Follow-up
TIMEPOINT**	*Preop visit*	Before GA	*Intraoperatively*	*PACU*	*POD 1*	*POD 2*	*POD X*	*Hospital discharge*	*POD30*
**Patient enrollment**									
**Eligibility criteria**	X								
**Written informed consent**	X								
Demographic data	X								
Baseline characteristics	X								
**Randomization/ Allocation**		X							
**Study interventions:**									
OFA-OBA-no preference choice		X							
OFA: opioid-free-anesthesia			X						
OBA: opioid-based-anesthesia			X						
**Outcome assessment:**									
QOR-15					◆—	—◆			
PONV				◆—	—	—	—	—◆	
Patient satisfaction					X				
Opioid requirement	X			◆—	—	—	—	—	—◆
DAH30									X
Factors associated with choice	X		X						
Surgical and anesthesia characteristics			X						
Intraoperative data and complications			X						
Postoperative pain scores				◆—	—	—	—	—◆	
Length of PACU stay				X					
Length of hospital stay								X	
EQ-5D-5L									X
Postoperative complications									X
Readmission rate									X

According to SPIRIT statement of defining standard protocol items for clinical trials.
DAH30: Days at home 30; EQ-5D-5L: EuroQol 5 Dimension, five-level version with visual analogue scale; GA: general anesthesia; PACU: postoperative care unit; POD: postoperative day; PONV: postoperative nausea and vomiting; QoR-15: quality of recovery 15.

WHAT ARE THE RISKS I SHOULD EXPECT? (Detailed Description)

Known risks and discomforts:

The possible risks and/or discomforts associated with the procedures described in this consent form include:

- Offering the possibility to choose your anesthesia strategy (or not) does not pose any risk. The study's exclusion criteria ensure that you can safely receive either OFA or OBA for your anesthesia.
- We expect the risks associated with being in the OFA group to be relatively low because your anesthesiologist can choose among five drugs (at least 2 drugs)

based on their clinical experience and your medical history. The anesthesiologist is free to decide which drugs to use. As a reference, the FDA approves the use of these five adjuvants during general anesthesia.

- Magnesium and dexmedetomidine may affect your blood pressure and heart rate (low blood pressure and slower heart rate) during anesthesia. Dose regimens have been adapted in the current protocol to limit these side effects. Additionally, these events typically occur in daily practice when you are under general anesthesia whatever OFA or OBA strategies. The protocol includes guidelines to manage these potential side effects, specifically hypotension and bradycardia.
  Anesthetic monitoring modalities including pulse oximetry, electrocardiography, noninvasive blood pressure and/or invasive monitoring to track theses potential effects.
- OFA does not affect your operation time, surgery and care after surgery.
- OBA may result in more nausea and vomiting compared to OFA. To address this, both groups will receive IV medications pre-emptively in attempt to reduce nausea, particularly during the first few hours after surgery is complete.
- There is a rare risk of breach in confidentiality related to the data collection.

Unknown risks and discomforts:
The experimental treatments may have side effects that no one knows about yet. The researchers will let you know if they learn anything that might make you change your mind about participating in the study.

HOW WILL INFORMATION ABOUT ME AND MY PARTICIPATION BE KEPT CONFIDENTIAL?

The researchers will do their best to make sure that your private information is kept confidential. Information about you will be handled as confidentially as possible, but participating in research may involve a loss of privacy and the potential for a breach in confidentiality. Study data will be physically and electronically secured. As with any use of electronic means to store data, there is a risk of breach of data security.

Use of personal information that can identify you:
All identifiable information about you will be replaced with a code. The link to the code and your identifiable information will be kept separate from the study data.

How information about you will be stored:
Study data will be physically and electronically secured. Physical data will be kept in a locked room with limited access by authorized personnel. Electronic data will be encrypted, or password protected and stored in a secure database and will be accessible only to study personnel.

People and agencies that will have access to your information:

The research team, authorized UCLA personnel, the study sponsor, and regulatory agencies such as the Food and Drug Administration (FDA), may have access to study

data and records to monitor the study. Research records provided to authorized, non-UCLA personnel will not contain identifiable information about you. Publications and/or presentations that result from this study will not identify you by name.

Employees of the University may have access to identifiable information as part of routine processing of your information, such as lab work or processing payment. However, university employees are bound by strict rules of confidentiality.

How long information from the study will be kept:
Data of the study should be kept for 15 years after the completion of the study.

USE OF DATA AND SPECIMENS FOR FUTURE RESEARCH

My data, including de-identified data can be kept for use in future research.

ARE THERE ANY COSTS FOR TAKING PART IN THIS STUDY?

There will be no additional cost to you or your health plan as a result of your participation in this study. Items and services described in this consent form would have occurred regardless of your participation in this study or, if research-related, will be provided to you at no cost.

WILL I BE PAID FOR MY PARTICIPATION?

You will not be paid for your participation in this research study.

WHO CAN I CONTACT IF I HAVE QUESTIONS ABOUT THIS STUDY?

The Research Team:
You may contact Dr. Alexandre Joosten at (310) 962-7337 with any questions or concerns about the research or your participation in this study. You can also call the UCLA Page Operator at (310) 825-6301 to reach Drs. Joosten 24 hours a day, 7 days week.

UCLA Office of the Human Research Protection Program (OHRPP):
If you have questions about your rights while taking part in this study, or you have concerns or suggestions and you want to talk to someone other than the researchers about the study, you may contact the UCLA OHRPP by phone: (310) 206-2040; by email: participants@research.ucla.edu or U.S. mail: UCLA OHRPP, Box 951406, Los Angeles, CA 90095-1406.

Public Information about this Study:
ClinicalTrials.gov is a website that provides information about federally and privately supported clinical trials. A description of this clinical trial will be available on http://www.ClinicalTrials.gov, as required by U.S. Law. This website will not include information that can identify you. At most, the website will include a summary of the results.

WHAT HAPPENS IF I BELIEVE I AM INJURED BECAUSE I TOOK PART IN THIS STUDY?

It is important that you promptly tell the researchers if you believe that you have been injured because of taking part in this study. You can tell the researcher in person or call him/her at the number(s) listed above.

If you are injured as a result of being in this study, UCLA will provide necessary medical treatment. The costs of the treatment may be covered by UCLA or billed to you or your insurer just like other medical costs, depending on a number of factors. The University and the study sponsor do not normally provide any other form of compensation for injury. For more information about this, you may call the UCLA Office of the Human Research Protection Program at (310) 206-2040 or send an email to participants@research.ucla.edu.

WHAT ARE MY RIGHTS IF I TAKE PART IN THIS STUDY?

Taking part in this study is your choice. You can choose whether or not to participate. Whatever decision you make, there will be no penalty to you, and you will not lose any of your regular benefits.

- You have a right to have all of your questions answered before deciding whether to take part.
- Your decision will not affect the medical care you receive from UCLA.
- If you decide to take part, you can leave the study at any time.
- If you decide to stop being in this study you should notify the research team right away. The researchers may ask you to complete some procedures in order to protect your safety.
- If you decide not to take part, you can still get medical care from UCLA.

HOW DO I INDICATE MY AGREEMENT TO PARTICIPATE?

If you want to participate in this study, you should sign and date below. You have been given a copy of this consent form and the Research Participant's Bill of Rights to keep. You will be asked to sign a separate form authorizing access, use, creation, or disclosure of health information about you.

SIGNATURE OF THE PARTICIPANT

____
Name of Participant

______________________________________ ______________________
Signature of Participant Date

SIGNATURE OF PERSON OBTAINING CONSENT

______________________________________ ______________________
Name of Person Obtaining Consent Contact Number

______________________________________ ______________________
Signature of Person Obtaining Consent Date
